# Registration based assessment of femoral torsion for rotational osteotomies based on the contralateral anatomy

**DOI:** 10.1186/s12891-022-05941-2

**Published:** 2022-11-08

**Authors:** Armando Hoch, Julian Hasler, Pascal Schenk, Jakob Ackermann, Lars Ebert, Philipp Fürnstahl, Patrick Zingg, Lazaros Vlachopoulos

**Affiliations:** 1 Department of Orthopaedics, Balgrist University Hospital, University of Zurich, Forchstrasse 340, 8008 Zurich, Switzerland; 2 Zurich Institute of Forensic Medicine, University of Zurich, Zurich, Switzerland; 3 Research in Orthopaedic Computer Science, Balgrist University Hospital, University of Zurich, Zurich, Switzerland

**Keywords:** 3D imaging, Computer assisted surgery, Femoral torsion, Femoral osteotomy

## Abstract

**Background:**

Computer-assisted techniques for surgical treatment of femoral deformities have become increasingly important. In state-of-the-art 3D deformity assessments, the contralateral side is used as template for correction as it commonly represents normal anatomy. Contributing to this, an iterative closest point (ICP) algorithm is used for registration. However, the anatomical sections of the femur with idiosyncratic features, which allow for a consistent deformity assessment with ICP algorithms being unknown. Furthermore, if there is a side-to-side difference, this is not considered in error quantification.

The aim of this study was to analyze the influence and value of the different sections of the femur in 3D assessment of femoral deformities based on the contralateral anatomy.

**Material and methods:**

3D triangular surface models were created from CT of 100 paired femurs (50 cadavers) without pathological anatomy. The femurs were divided into sections of eponymous anatomy of a predefined percentage of the whole femoral length. A surface registration algorithm was applied to superimpose the ipsilateral on the contralateral side. We evaluated 3D femoral contralateral registration (FCR) errors, defined as difference in 3D rotation of the respective femoral section before and after registration to the contralateral side. To compare this method, we quantified the landmark-based femoral torsion (LB FT). This was defined as the intra-individual difference in overall femoral torsion using with a landmark-based method.

**Results:**

Contralateral rotational deviation ranged from 0° to 9.3° of the assessed femoral sections, depending on the section. Among the sections, the FCR error using the proximal diaphyseal area for registration was larger than any other sectional error. A combination of the lesser trochanter and the proximal diaphyseal area showed the smallest error. The LB FT error was significantly larger than any sectional error (*p* < 0.001).

**Conclusion:**

We demonstrated that if the contralateral femur is used as reconstruction template, the built-in errors with the registration-based approach are smaller than the intraindividual difference of the femoral torsion between both sides. The errors are depending on the section and their idiosyncratic features used for registration. For rotational osteotomies a combination of the lesser trochanter and the proximal diaphyseal area sections seems to allow for a reconstruction with a minimal error.

## Introduction

Computer-assisted techniques for the surgical treatment of femoral deformities have become increasingly important [1–9]. These techniques allow three-dimensional (3D) assessment of deviations from the normal anatomy in all anatomical planes and enable 3D preoperative planning. Along with patient specific instruments or intraoperative navigation they enable the surgeon to perform an extremlysophisticated correction and thereby restoring the physiological anatomy. It was shown that the 3D correction planned on a computer can be implemented accurately in the surgery [8, 9]. For example, a multiplanar deformity of the proximal femur with the necessity for intraarticular correction can be reliably assessed, enablingsurgery to be performed accordingly [2].

In computer-assisted reconstructive surgery, the state-of-the-art technique for 3D deformity assessment relies on a reconstruction template representing the normal anatomy. The two established methods used are the application of a statistical shape model of an average anatomy or the utilization of a model of the contralateral side of the treated individual [10–15]. As there are relevant inter-ethnic differences and a statistical shape model is not always available, the use of the contralateral anatomy has gained acceptance [16–18]. This accommodates a surface registration method to be applied to superimpose the model of the pathological side onto the mirrored model of the contralateral side [8, 10–15]. In the femur, the proximal (trochanteric region) and the distal (femoral condyles) anatomy are typically used to achieve the overlay of the two models [2, 8]. As in conventional planning with sectional imaging, the deformity is then assessed for the entire femur as one single value (i.e. torsional error in degrees) [19, 20]. The quantification of the deformity based on the template of the contralateral side might be especially error prone, when there are side-to-side differences between the side to be corrected and the contralateral template. This problem was recently described and a novel registration-based approach relying on the presence of idiosyncratic features of long bones (i.e. humerus) was introduced to compensate for bilateral differences of the anatomy [13, 14]. However, it was noted that the consistency of registration would need to be validated separately when considering implementing this new approach for other skeletal sites (i.e. femur) as the idiosyncratic features that are involved would be different.

The aim of this study was to analyze the influence and value of the different three-dimensional sections of the femur in 3D registration-based assessment of the femoral deformities based on the contralateral anatomy according to the very similar methodology for humeral deformities of Vlachopoulos et al [13, 14]. Finally, we aimed to suggest the section to be used for registration in rotational osteotomies of the femur in order to incorporate the smallest possible error. Thus, for subtrochanteric rotational osteotomies, the question is: Which contralateral section of the femur must be used for registration for the orientation of the section proximal to the osteotomy plane to undergo the smallest possible change?

## Material and methods

The Zurich Institute of Forensic Medicine (IRMZ, University of Zurich, Zurich) provided anonymized post mortem computed tomography (PMCT) data of both femurs of 50 cadavers without femoral pathology. In all 50 cadavers the full length of the femur was available. The scans were performed on a Somatom Definition Flash CT scanner (Siemens Healthineers, Erlangen, Germany) using a standardized protocol [21]. The resolution of the CT scans was 512 × 512 pixels. The slice thickness was 1.0 mm in all scans. The mean age of the individuals was 57 years (range 18–86 years). There were 35 male and 15 female cadavers. The mean height was 172 cm (range 137–190 cm) and the mean weight was 71 kg (range 41–110 kg). The exclusion criteria were post-operative or post-traumatic alterations of the bony anatomy or any signs of bony malformations. The local ethical committee approved this study (Zurich Cantonal Ethics Commission, 2017–01616) and all patients gave their informed consent.

The CT scans were segmented using the global thresholding and region growing functionality of a standard segmentation software (Mimics Medical, Materialise NV, Leuven, Belgium) in order to generate 3D bone models [22–24]. Thereafter, the models were imported into the planning software CASPA (Computer Assisted Surgery Planning Application) that was developed in-house (Balgrist ROCS [Research in Orthopaedic Computer Science]).

### Current state-of-the-art assessment for rotational osteotomies

Various techniques can be used to assess a pathologically altered rotation of the femur. For a unilateral (e.g. post-traumatic) deformity, the analysis is often performed based on the contralateral side. These assessments can be carried out in two or three dimensions. Either the measurement is carried out as a projected 2D measurement in sectional imaging or a 3D measurement is performed using the entire volumetric data set. The main benefit of 3D measurement is the accurate quantification of multiplanar deformities.

Regardless of the technique, the proximal and distal parts of the femur (femoral neck, condyles) are used to calculate the rotation, especially as anatomically distinctive landmarks are available for orientation on these parts of the bone. Both techniques result in appliance of the full amount of the rotational difference for correction of the pathological femur at the level of the osteotomy, with no consideration of the preexisting rotation distributed over the entire femur.

### Effect of segment selection on rotational assessment

For registration of the models an ICP (iterative closest point) algorithm was used. The principle of the ICP algorithm is to superimpose the source model onto the target model in such a way that the difference between the two models is as minimal as possible within the selected anatomical 3D section [25]. It was previously demonstrated on the humerus, that the selection of sections with the same structural characteristics as close as possible to the pathological area is relevant for registration-based techniques to compensate for bilateral differences [13, 14]. The further away the registered distal part of the humerus from the plane of correction, the greater the built-in the error. However, the consistency of registration has to be validated separately for further anatomies (i.e. femur).

**Fig. 1:**
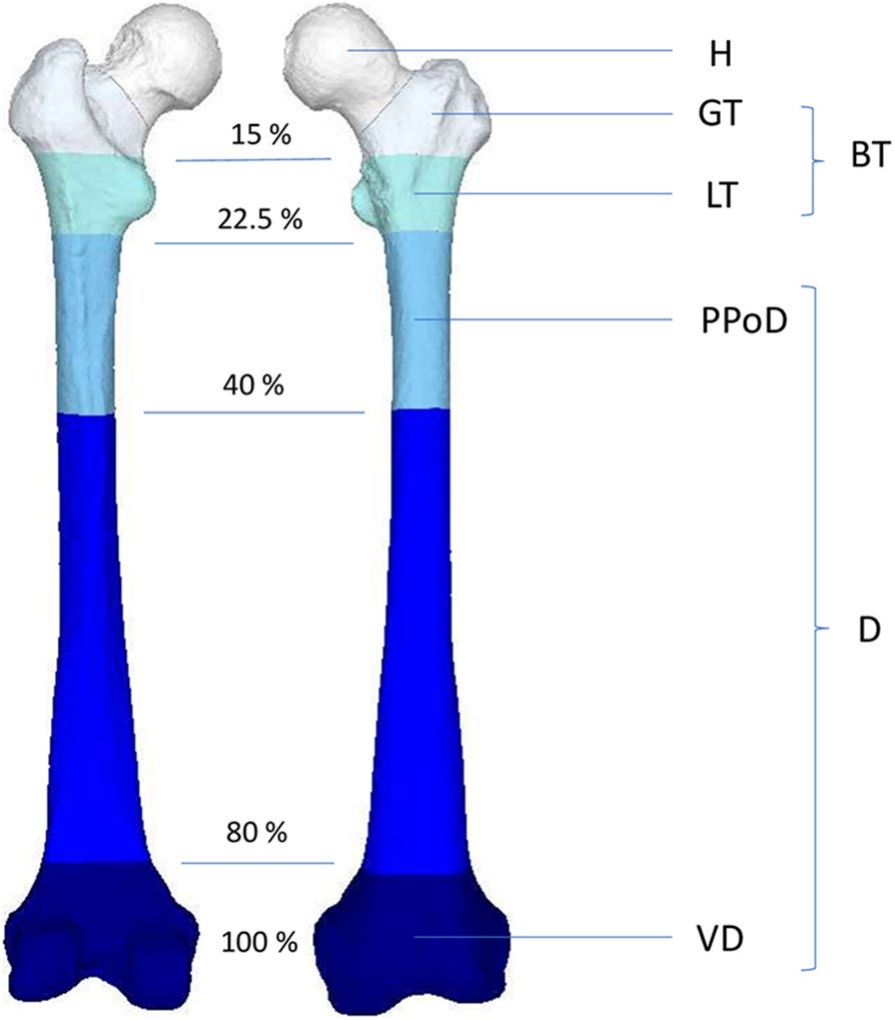
Predefined Sections of the Femur with Percentage of the Whole Femur (H: Head, GT: Greater Trochanter, BT: Both Trochanters, LT: Lesser Trochanter, PPoD: Proximal Part of Distal, D: Distal, VD: Very Distal)

In the present study, we used bilateral femoral models without a pathological condition as this is the only way to analyze the effect of segment selection on the assessment of femoral torsion and the hypothetical correction based on it. In an ideal case, the torsional differences of the single corresponding sections should be zero. In order to assess the distribution of the torsional error between the two femurs we divided one femur into predefined sections and registered in on the contralateral femur with the ICP algorithm as previously described [13, 14]. The sections were selected in such a way that the eponymous anatomical landmarks were included in the respective section and the following percentages of sections regarding the entire femur could be fulfilled: GT (Greater Trochanter): Proximal 15%, LT (Lesser Trochanter: 7.5% distal to GT, BT (Both Trochanters): Proximal 22.5%, PPoD (Proximal Part of Diaphysis): 17.5% distal to LT, D (Distal): Distal 80%, VD (Very Distal): Distal 20%. The predefined sections are depicted in Fig. 1.

**Fig. 2:**
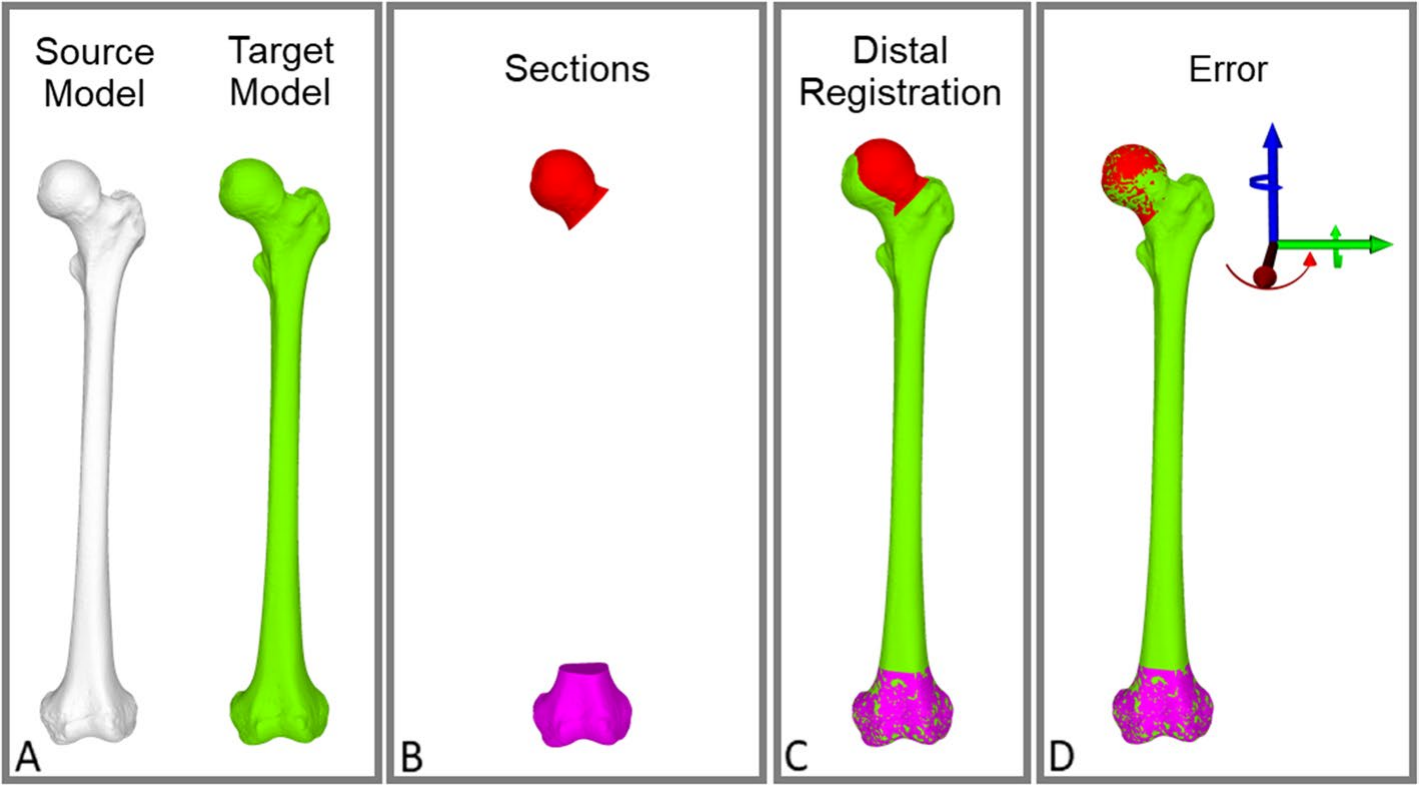
Registration Process. **A**: Source and Target Model, **B**: Sections of Interest (Red: Head Section, Purple: Very Distal Section. **C**: Registration of Very Distal Source Region on Target Model, **D**: Registration of Head Source on Target Model for Error Quantification)

The deformity was assessed as follows (see Fig. 2): The femur of the contralateral side was mirrored in order to serve as reconstruction template. The two femoral models (ipsi and contralateral, following called ‘source’ and ‘target’ model) were first coarsely superimposed. Then, each of the previously mentioned distal sections (GT, LT, BT, LT and PPoD, PPoD, D, VD) of the source model was used to superimpose the source model on the target model using the ICP algorithm [25, 26], hereafter called distal registration. For the quantification of the rotational difference between both sided we defined the femoral head (H) segment. This H section was defined as the part of the femur proximal to a cut through the femoral neck performed 1.25 times the radius of the femoral head distal to its center. This value was chosen in order to not accidently integrate parts of the greater trochanter into the H segment. The difference in the orientation of the femoral head segment between the source and the target model reflect the rotational difference between both sides if the previous described distal segment was used for the distal registration (Fig. 2c). For the quantification of the deformity we used the ICP algorithm again to superimpose the H segment to the target model (proximal registration). The difference of the H segment before and after the proximal registration is called femoral contralateral registration (FCR) error. It can be described as a 3D angle (3D FCR) in axis-angle representation or as 3 consecutive rotations in all three anatomical planes. A 3D FCR error of zero degrees indicates that there were no torsional side-to-side differences if the selected distal segment was used for registration and in the case of a rotational osteotomy there would not be any built-in error if the respective section is used for registration in the planning of the surgery. All measurements were performed with respect to the standardized coordinate system of the International Society of Biomechanics (ISB) [27]. The rotation around the x-axis corresponds to a rotation in the axial plane and represents the torsional differences between both sides. The x axis was defined using the longitudinal axis of the smallest possible square (or oriented bounding box) that can enclose a volumetric model.

First, the right femur was defined as source model. The left femur was mirrored and served as the target model. All experiments were repeated using the right femur as source and the left femur as target model. This allowed the assessment of the consistency of the method using the intraclass correlation coefficient (ICC) in order to exclude a bias introduced through the unilateral selection of target and source model. For a total of 7 sections in 50 individuals, this led to 700 registration experiments.

### Landmark-based method

**Fig. 3:**
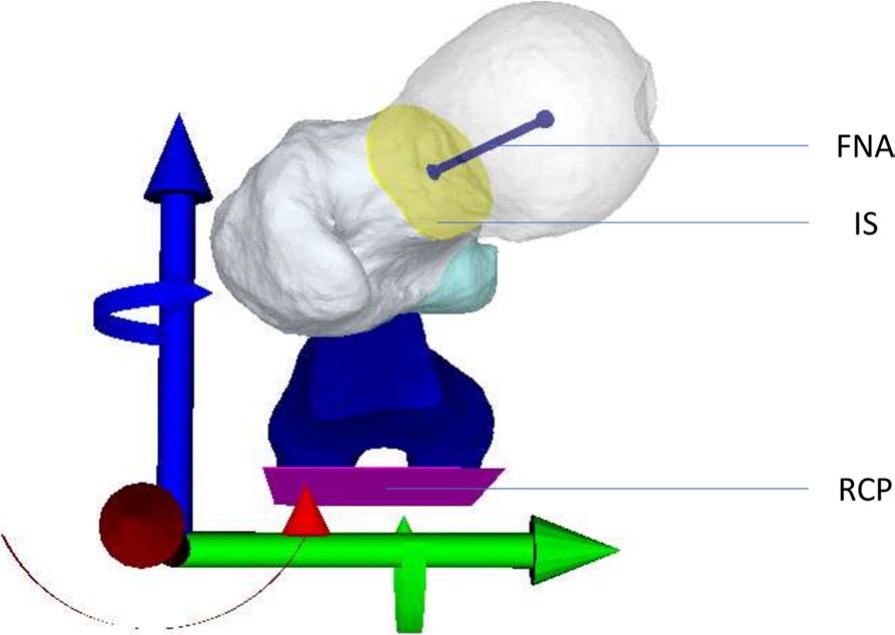
Measurement of Landmark-Based Femoral Torsion (FNA: Femoral Neck Axis, IS: Intersection, RCP: Retrocondylar Plane)

For comparison of the FCR errors based on the bilateral differences in femoral torsion, we assessed the landmark-based femoral torsion (LB FT) error. We measured the torsion of each femur between the femoral neck axis (FNA) and the retrocondylar plane (RCP) (Fig. 3). For definition of the FNA, the center of the femoral head (FHC) was defined by the center of a sphere fitted to the surface of the femoral head. A cylinder bound to this center was aligned into the femoral neck. We defined a plane with the FNA as plane normal and a distance of 1.25 times the femoral head radius from the FHC. The center of mass of the intersection (IS) between the femoral model and this plane was defined as the center of the femoral neck (FNC). The FNA was then defined as the vector between the FNC and the FHC. For definition of the RCP, an oriented bounding box of the femur was rotated around its longitudinal axis and adjusted in its anteroposterior expansion until the most posterior points on the medial and lateral femoral condyles were located on the surface of the box. The RCP was defined by the normal of this plane touching the posterior points (Fig. 3). The FNA and the RCP vectors were projected onto the y-z plane of the femoral coordinate system. The LB FT error was the absolute difference in the torsion between both sides of an individual. The portion of the 3D FCR error around the x-axis can be interpreted as femoral torsion and is comparable with the LB FT error.

### Statistical analysis

Interobserver reliability was assessed with the ICC for each measurement using a two-way mixed model for calculation. The assumption of sphericity was tested utilizing the Mauchly sphericity test. As the assumption of sphericity was violated, we accordingly applied the nonparametric Friedman rank-sum test with the segment as a group factor and the individuals as a block factor to analyze the effect of the defined segments on the FCR error. Post hoc analysis was performed using the Wilcoxon signed-rank test with adjusted Bonferroni *p*-values. For graphical FCR error visualization, Tukey boxplots were used, with the ends of the whiskers indicating 1.5 times the interquartile range (IQR) between the lower and upper quartiles, and outliers denoted with a circle. Descriptive statistics of the rotational errors were calculated from the absolute values. All statistical analyses were performed in SPSS for Mac (Version 23.0, SPSS Inc., Chicago, Illinois). Significance was set at *p* < 0.05.

## Results

### Intraclass correlation for rotational measurements

Absolute measurements of total femoral anteversion and each femoral section showed good to excellent reliability between readers (0.881–0.997, *p* < 0.001) (Table 1). Similar ICCs were seen for coronal and sagittal rotation measurements (0.852–0.997, *p* < 0.001) (Tables 2 and 3).

**Table 1:** Intraclass correlation coefficients (ICC) with 95% confidence intervals for anteversion measurements for each side

	ICC	95% CI	p		ICC	95% CI	p
**Ante Left**	0.994	0.990–0.997	< 0.001	**Ante Right**	0.993	0.988–0.996	< 0.001
**GT Left**	0.977	0.959–0.987	< 0.001	**GT Right**	0.927	0.872–0.959	< 0.001
**LT Left**	0.963	0.935–0.979	< 0.001	**LT Right**	0.946	0.905–0.969	< 0.001
**BT Left**	0.976	0.959–0.987	< 0.001	**BT Right**	0.881	0.790–0.932	< 0.001
**LT and PPoD Left**	0.993	0.988–0.996	< 0.001	**LT and PPoD Right**	0.890	0.808–0.938	< 0.001
**PPoD Left**	0.986	0.975–0.992	< 0.001	**PPoD Right**	0.937	0.890–0.964	< 0.001
**D Left**	0.990	0.982–0.994	< 0.001	**D Right**	0.941	0.897–0.967	< 0.001
**VD Left**	0.997	0.995–0.998	< 0.001	**VD Right**	0.944	0.901–0.968	< 0.001

**Table 2:** Intraclass correlation coefficients (ICC) with 95% confidence intervals for coronal rotation measurements for each side

	ICC	95% CI	p		ICC	95% CI	p
**GT Left**	0.956	0.922–0.975	< 0.001	**GT Right**	0.852	0.740–0.916	< 0.001
**LT Left**	0.969	0.946–0.983	< 0.001	**LT Right**	0.997	0.996–0.999	< 0.001
**BT Left**	0.968	0.944–0.982	< 0.001	**BT Right**	0.941	0.897–0.967	< 0.001
**LT and PPoD Left**	0.970	0.947–0.983	< 0.001	**LT and PPoD Right**	0.998	0.996–0.999	< 0.001
**PPoD Left**	0.950	0.912–0.072	< 0.001	**PPoD Right**	0.931	0.878–0.961	< 0.001
**D Left**	0.956	0.923–0.975	< 0.001	**D Right**	0.972	0.950–0.984	< 0.001
**VD Left**	0.938	0.891–0.965	< 0.001	**VD Right**	0.993	0.988–0.996	< 0.001

**Table 3:** Intraclass correlation coefficients (ICC) with 95% confidence intervals for sagittal rotation measurements for each side

	ICC	95% CI	p		ICC	95% CI	p
**GT Left**	0.991	0.983–0.995	< 0.001	**GT Right**	0.957	0.925–0.976	< 0.001
**LT Left**	0.983	0.969–0990	< 0.001	**LT Right**	0.984	0.971–0.991	< 0.001
**BT Left**	0.985	0.974–0.992	< 0.001	**BT Right**	0.953	0.918–0.974	< 0.001
**LT and PPoD Left**	0.992	0.985–0.995	< 0.001	**LT and PPoD Right**	0.979	0.962–0.988	< 0.001
**PPoD Left**	0.992	0.986–0.996	< 0.001	**PPoD Right**	0.981	0.967–0.989	< 0.001
**D Left**	0.989	0.980–0.994	< 0.001	**D Right**	0.978	0.961–0.987	< 0.001
**VD Left**	0.990	0.982–0.994	< 0.001	**VD Right**	0.968	0.943–0.982	< 0.001

### Restoration of the femur

**Fig. 4:**
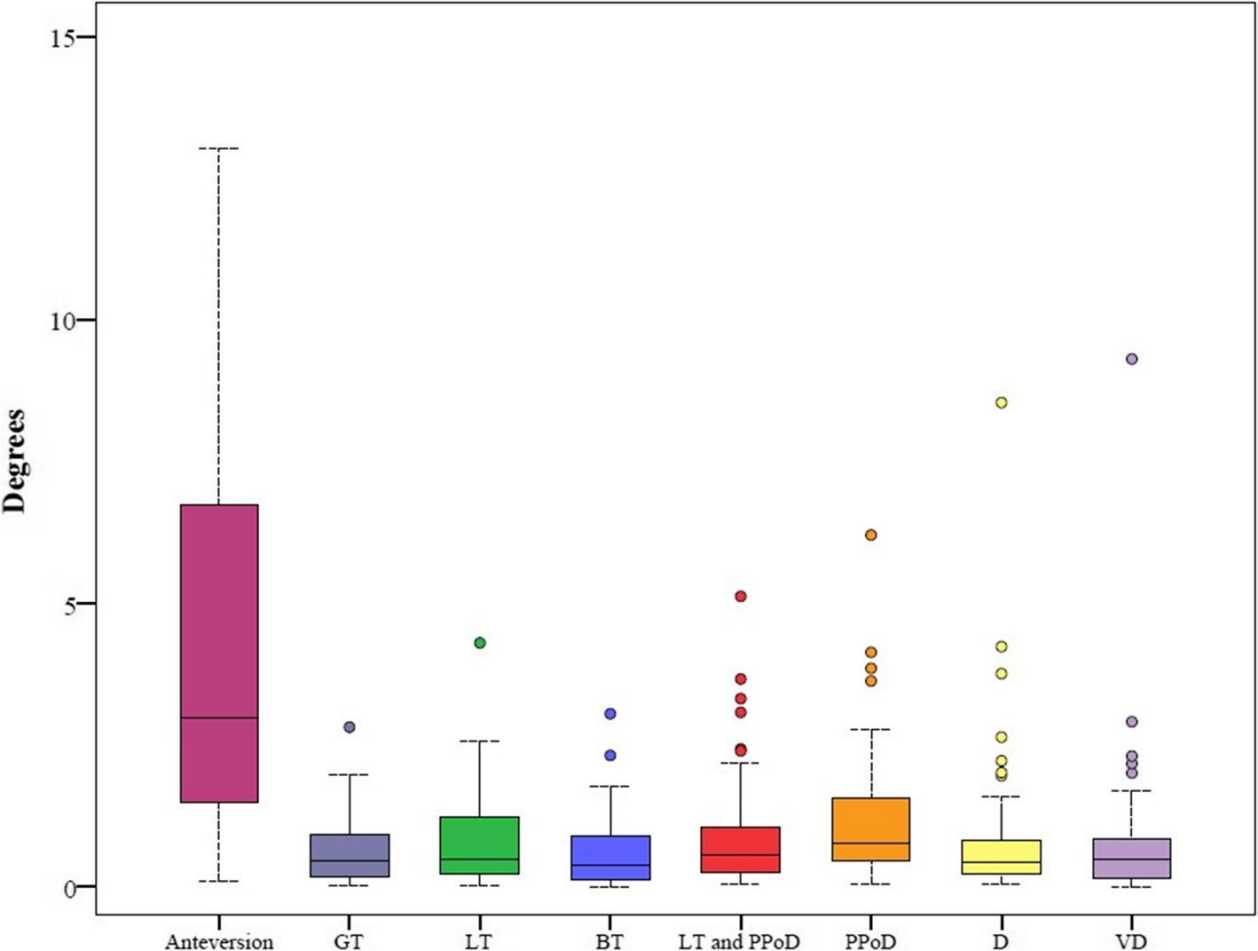
Rotational Error (GT: Greater Trochanter, BT: Both Trochanters, LT: Lesser Trochanter, PPoD: Proximal Part of Distal, D: Distal, VD: Very Distal)

Contralateral rotational deviation ranged from 0.1° to 13° for LB FT and 0° to 9.3° for the assessed femoral sections, depending on the section. The LB FT error was significantly larger than any sectional error (*p* < 0.001). Among the sections, the FCR PPoD error was significantly larger than both the FCR GT and BT errors (both, *p* < 0.001) (Fig. 4 and Table 4).

**Table 4:** Comparison of Anteversion Errors in the Contralateral Registration Modeling with an adjusted Bonferroni *p*-value of 0.00178. The asterisks indicate statistical significance

	Rotation Error (in degrees)				***P***-Values						
Segment	Mean	SD	Median	Range	FCR Error GT	FCR Error LT	FCR Error BT	FCR Error LT and PPoD	FCR Error PPoD	FCR Error D	FCR Error VD
**FCR Error Ante**	4.0	3.1	3.0	0.1–13.0	< 0.001*	< 0.001*	< 0.001*	< 0.001*	< 0.001*	< 0.001*	< 0.001*
**FCR Error GT**	0.6	0.6	0.5	0–2.8		0.062	0.761	0.033	< 0.001*	0.562	0.973
**FCR Error LT**	0.8	0.8	0.5	0–4.3			0.034	0.201	0.003	0.342	0.172
**FCR Error BT**	0.6	0.6	0.4	0–3.1				0.007	< 0.001*	0.352	0.851
**FCR Error LT and PPoD**	1.0	1.1	0.6	0.1–5.1					0.026	0.499	0.181
**FCR Error PPoD**	1.3	1.2	0.8	0–6.2						0.031	0.002
**FCR Error D**	0.9	1.4	0.4	0–8.5							0.746
**FCR Error VD**	0.8	1.4	0.5	0–9.3							

**Fig. 5:**
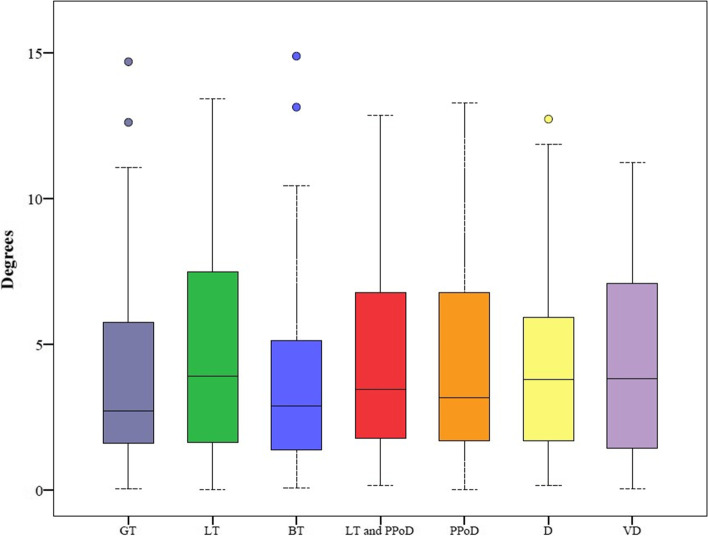
Coronal Error (GT: Greater Trochanter, BT: Both Trochanters, LT: Lesser Trochanter, PPoD: Proximal Part of Distal, D: Distal, VD: Very Distal)

**Fig. 6:**
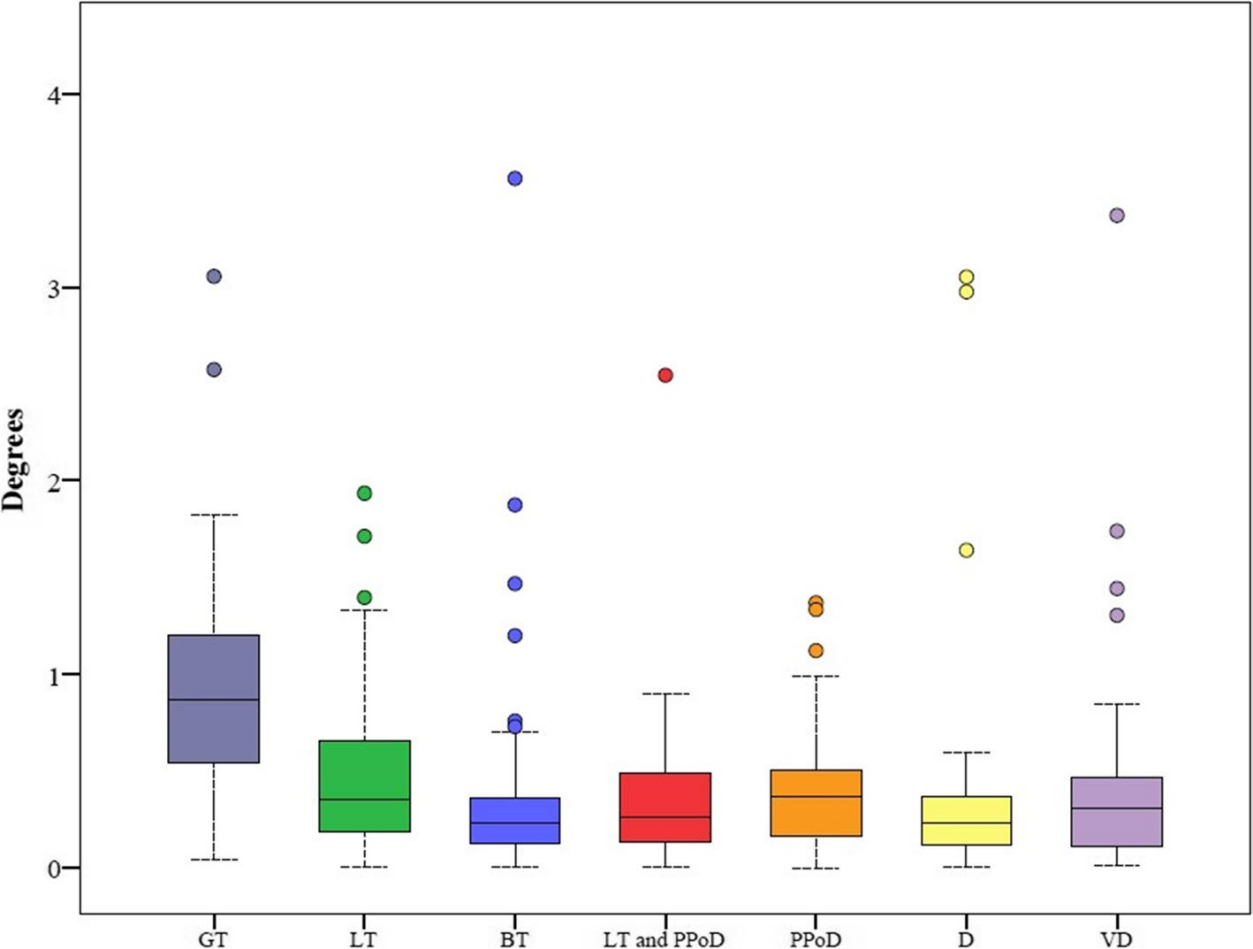
Sagittal Error (GT: Greater Trochanter, BT: Both Trochanters, LT: Lesser Trochanter, PPoD: Proximal Part of Distal, D: Distal, VD: Very Distal)

Contralateral coronal (varus / valgus) rotational deviation ranged from 0.0° to 3.5°, depending on the section. The FCR GT error was significantly larger than any other sectional error (*p* < 0.001) (Fig. 5 and Table 5). Sagittal (flexion / extension) rotational errors ranged from 0.0° to 14.9°, depending on the section. No statistically significant difference was seen between the sectional errors (*p* = 0.108) (Fig. 6 and Table 6).

**Table 5:** Comparison of Coronal Errors in the Contralateral Registration Modeling with an adjusted Bonferroni *p*-value of 0.00238. The asterisks indicate statistical significance

Rotation Error (in degrees)					***P***-Values					
Segment	Mean	SD	Median	Range	FCR Error LT	FCR Error BT	FCR Error LT and PPoD	FCR Error PPoD	FCR Error D	FCR Error VD
**FCR Error GT**	0.9	0.6	0.9	0.0–3.1	< 0.001*	< 0.001*	< 0.001*	< 0.001*	< 0.001*	< 0.001*
**FCR Error LT**	0.5	0.4	0.4	0.0–2.0		0.06	0.132	0.787	0.010	0.172
**FCR Error BT**	0.4	0.6	0.2	0.0–3.5			0.647	0.076	0.896	0.431
**FCR Error LT and PPoD**	0.4	0.4	0.3	0.0–2.5				0.039	0.068	0.689
**FCR Error PPoD**	0.4	0.3	0.4	0.0–1.4					0.001*	0.208
**FCR Error D**	0.4	0.6	0.2	0.0–3.1						0.062
**FCR Error VD**	0.4	0.6	0.3	0.0–3.4

**Table 6:** Comparison of Sagittal Errors in the Contralateral Registration Modeling. Friedman-sum rank test revealed no statistical significance between segments (*p* = 0.108)

Rotation Error (in degrees)					***P***-Values						
	Mean	SD	Median	Range	FCR Error GT	FCR Error LT	FCR Error BT	FCR Error LT and PPoD	FCR Error PPoD	FCR Error D	FCR Error VD
**FCR Error GT**	4.0	3.6	2.7	0.1–14.7							
**FCR Error LT**	4.7	3.9	3.9	0.0–13.4		No statistical significance between segments					
**FCR Error BT**	3.9	3.5	2.9	0.1–14.9							
**FCR Error LT and PPoD**	4.6	3.7	3.4	0.2–12.9							
**FCR Error PPoD**	4.6	3.6	3.2	0.0–13.3							
**FCR Error D**	4.4	3.3	3.8	0.2–12.7							
**FCR Error VD**	4.5	3.4	3.8	0.1–11.2							

## Discussion

In the present study, we quantified and localized the side-to-side difference in bilateral femurs of 50 cadavers. The mean LB FT error was 4.0° (range 0.1° - 13.0°), which corresponds to the intraindividual side-to-side difference of the femoral torsion. This finding is in line with the actual literature [28]. According to current planning methods, this difference is not considered in the planning. We showed that this torsional difference is not homogenously distributed over the entire femur. Interestingly, this is in contrast to the results on the humerus. There, a different result was achieved with a very similar methodology. The error was smaller, when a segment close to the humeral head was used. The assumption from the experience of Vlachopoulos et al. must therefore not be transferred to the femur [13, 14]. However, for the proximal femur, the registration of the proximal part of the diaphysis (PPoD, mean 1.3°) is more prone to error than further distal sections. When the section of the greater trochanter (GT, mean 0.6°) or both trochanters (BT, mean 0.6°) are used for registration, the torsional difference is small and when the very distal (VD, mean 0.8°) section is used, the difference is moderate.

This has some relevant clinical implications. Thus, even if there is no side-to-side difference or the correction is performed to a predefined value, it is important to note that the appliance of a correction in the sense of a rotational osteotomy at a certain level (e.g. subtrochanteric osteotomy), one should consider the preexisting torsion of the femur. When using the contralateral femur as reconstruction template, the built-in errors are depending on the section and their idiosyncratic features used for registration. For example for a rotational subtrochanteric osteotomy, where of course only sections below the trochanters can be used for registration, the very distal section with its femoral condyles allows for a reconstruction with a minimal error (0.8°). If the VD section is used, the built-in error is substantially smaller compared to a correction to a standard value or the LB FT (4.0°) error. We were able to show that a minimalization of the built-in error can be achieved, if the section of the femur that is used for registration, is selected with prudence. The used proposed approach for contralateral registration in this work allows for this which was demonstrated with a very good repeatability and high ICC.

The errors in the sagittal and coronal plane introduced after registration have also to be considered. The errors in a coronal plane, leading to varus or valgus deformity are relatively small. The errors that occur during contralateral registration in the sagittal plane are larger. This might be less problematic if only a correction of the femoral torsion is planned. However, if an additional correction of a coronal or sagittal deformity is planned, this might be more problematic.

This study has some limitations. The methodology was studied on healthy femurs without pathology. This has the disadvantage that an application for the actual goal of this registration-based method is missing. Nevertheless, the underlying question, namely what error would be incorporated if the contralateral side is used for registration, can only be answered in this way. Furthermore, the methodology is complex and requires, at least in its development, some expertise in computer assisted surgery. Nevertheless, we think that a large part of these steps can be automated, so that an implementation in the clinical routine is well possible. Furthermore, the insights gained also imply an adjustment of the conventional treatment of corresponding patients. Thus, a sensitization for the three-dimensionality of a problem takes place and one probably considers more precisely where an osteotomy should be performed.

We conclude that there is a relevant side-to-side difference between the torsion of the femurs in our collective and this torsion is inhomogenously distributed. When using the contralateral femur as reconstruction template, the built-in errors are depending on the section and their idiosyncratic features used for registration. For rotational subtrochanteric osteotomies to the very distal section allows for a reconstruction with a minimal error. This study enables to choose the ideal section for registration for every desired osteotomy level.

## Data Availability

The datasets used and/or analyzed during the current study are not publicly available due to the use of the data for another research project with a completely different research question but are available from the corresponding author on reasonable request.

## Abbreviations

2D: Two dimensional
3D: Three dimensional
BT: Both trochanters
cm: Centimeters
CT: Computed tomography
D: Distal
FCR: Femoral contralateral registration
FHC: Center of the femoral head
FNA: Femoral neck axis
GT: Greater trochanter
H: Head
ICC: Intraclass correlation coefficient
ICP: Iterative closest point
IS: Intersection
IQR: Interquartile range
kg: Kilograms
LB FT: Landmark-based femoral torsion
LT: Lesser trochanter
mm: Milimeters
PPoD: Proximal part of diaphysis
RCP: Retrocondylar plane
VD: Very distal
